# The Composition of Native Plant Species and Nitrogen Availability Jointly Influence the Invasion Success of *Cenchrus spinifex*

**DOI:** 10.3390/plants15132016

**Published:** 2026-06-29

**Authors:** Jiyun Yang, Long Yan, Chuan Lu, Haizhou Jiang, Xiaolin Sun, Baihui Ren, Yulong Feng

**Affiliations:** 1 College of Horticulture, Shenyang Agricultural University, Shenyang 110866, China; yangjiyun@syau.edu.cn (J.Y.); y17635217516@163.com (L.Y.); m18304054722@163.com (C.L.); dfvv123@163.com (H.J.); linuwb@163.com (X.S.); 2 Liaoning Key Laboratory for Biological Invasions and Global Changes, College of Bioscience and Biotechnology, Shenyang Agricultural University, Shenyang 110866, China

**Keywords:** biological invasion, nitrogen deposition, plant functional group, *Cenchrus spinifex*, rhizosphere microorganisms

## Abstract

Nitrogen deposition continuously alters the invasibility of terrestrial ecosystems, but how the composition of local plant functional groups regulates this process by root-associated microbial during invasion, especially under the background of resource changes, remains unclear. This study focused on the invasive plant *Cenchrus spinifex* Cav. and conducted an interactive experiment using nitrogen addition and four different functional group combinations of local plant communities. The results show that the community with the closest phylogenetic distance (PD = 189) had the strongest resistance to invasion. Nitrogen addition was the core factor driving invasion (total effect 0.86), which promoted invasion by increasing soil nitrogen pools and altering microbial community structure. The role of leguminous plants changed fundamentally with nitrogen availability; they were competitors under low-nitrogen conditions, while under high-nitrogen conditions, they transformed into “synergistic invaders” by shaping the root-associated environment rich in microorganisms such as Proteobacteria that facilitate rapid nutrient turnover. Plant nitrogen and phosphorus content (PNP) is a key indicator reflecting the nutrient absorption capacity of invasive plants and is closely related to invasion success. It significantly promotes the ability of root resources acquisition. The study shows that invasion success depends on the dynamic balance among resource input, the phylogenetic background of the local community, and the microbial feedback regulated by it. Future ecological management should consider the coordinated regulation of aboveground functional group selection and underground microbial processes.

## 1. Introduction

Biological invasion is an important component of global change, posing a serious threat to the biodiversity, structure and function of ecosystems [1]. The outcome of the invasion process is essentially a result of the interplay between the invasive ability of alien species and the resistance of local biological communities [2]. Among the many factors determining community resistance, the species composition and functional diversity of local plant communities are considered the intrinsic core [3]. The presence or absence of specific functional groups (such as nitrogen-fixing plants and allelopathic plants) may profoundly affect the invasibility of the community by altering the resource competition pattern or generating non-resource interactions [4]. Meanwhile, the continuous increase in global nitrogen deposition caused by human activities is changing the nitrogen cycle in terrestrial ecosystems at an unprecedented rate [5]. As a key environmental driver, nitrogen deposition can affect both invasive and native species by alleviating the widespread nitrogen limitation [6], altering soil chemistry and microbial processes [7], potentially weakening the resistance of local communities and creating an “invasion window”, thereby exacerbating biological invasion [8].

However, different functional groups and species respond significantly differently to nitrogen enrichment [8,9]. For instance, leguminous plants, with their symbiotic nitrogen-fixing ability, are less directly affected by fluctuations in soil nitrogen availability and may instead alter the nitrogen cycle and competitive balance within the community through biological nitrogen fixation [10,11]. In contrast, many non-nitrogen-fixing grass species are highly dependent on soil available nitrogen, and their competitive ability may significantly increase with nitrogen addition, especially in light resource competition [12,13]. Additionally, the allelopathic substances released by some *Asteraceae* plants may also change their inhibitory effects on neighboring plants in a nitrogen-rich environment [14]. Therefore, understanding how the functional composition of local plant communities differentially regulates their resistance to specific invasive species under the background of nitrogen enrichment is crucial for predicting future invasion dynamics and formulating targeted management strategies.

The key to determining whether an alien species can successfully invade lies not only in the degree of similarity in functional traits with local species [15], but also in their evolutionary relationship. The “limiting similarity hypothesis” posits that invaders with highly overlapping ecological niches with local species will face stronger competitive exclusion [16]. This hypothesis can be deepened from the perspective of phylogenetic ecology; species with close phylogenetic relationships often share similar functional traits and ecological niches [17]. Therefore, local communities that are phylogenetically closer to invasive species may exhibit stronger competition due to higher trait overlap, thereby demonstrating stronger resistance to invasion. Although the “diversity resistance hypothesis” posits that communities with high species diversity have stronger resistance to invasion [18], an increasing body of evidence suggests that, at the local scale, the phylogenetic structure of a community is often more crucial than the sheer number of species [17,18,19]. Currently, there is a lack of systematic experimental research on whether local plant communities with the same number of species but different functional group compositions and phylogenetic backgrounds have different resistances to invasive plants, what the mechanisms are, and especially how nitrogen deposition regulates this process.

*Cenchrus spinifex* Cav., a noxious invasive grass native to the Americas, has widely spread in northern China, severely disrupting the stability of grassland ecosystems and causing significant economic losses [20]. *C*. *spinifex* has a strong ability to absorb and utilize nitrogen and phosphorus [13], which may give it an advantage in competition with local plants. Its invasion can alter the structure of soil microbial communities [21], and nitrogen addition may further change its rhizosphere microenvironment, enriching microorganisms related to rapid nutrient turnover [22]. However, there is still a lack of systematic exploration of the resistance mechanisms of invaded local plant communities from the perspective of their functional composition and phylogenetic background.

Therefore, this study will interactively design nitrogen deposition and the functional composition of local plant communities, setting two levels of low- and high-nitrogen and four different functional group combinations. With the relative biomass ratio of *C*. *spinifex* as the core response variable, combined with phylogenetic distance analysis, plant growth and reproductive traits, soil nutrient and microbial community analysis, the study aims to explore (1) whether local plant communities with the same number of species but different functional group compositions and phylogenetic backgrounds have different resistances to the invasion of *C*. *Spinifex* (2) How does nitrogen addition regulate the above relationship by altering the rhizosphere microbial community (especially beneficial or harmful groups to the invasive species)? (3) How do phylogenetic distance, functional group identity, and rhizosphere microbial processes interact to jointly determine the invasion outcome? This study will provide multi-dimensional theoretical basis for constructing more predictive and targeted invasion prevention and control strategies under the background of nitrogen deposition.

## 2. Results

### 2.1. The Interactive Effects of Nitrogen Availability and Local Plant Species Composition on the Invasiveness of C. spinifex

**Figure 1:**
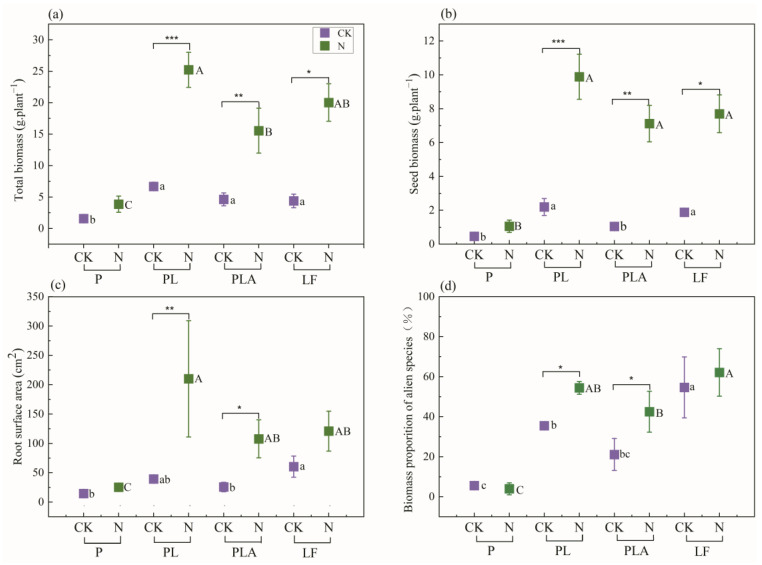
The effects of nitrogen addition and local plant species composition on the invasion ability of *C. spinifex*. (**a**) Total biomass, (**b**) seed biomass, (**c**) root surface area, and (**d**) biomass ratio of the *C. spinifex*. Different lowercase letters indicate significant differences among species combinations under the CK treatment; different uppercase letters indicate significant differences among species combinations under the nitrogen application treatment (*p* < 0.05); * (*p* < 0.05), ** (*p* < 0.01), and *** (*p* < 0.001) indicate the significance of differences in the same species combination under different N availabilities, as follows.

Nitrogen addition significantly promoted the total biomass, seed biomass, root surface area and biomass proportion of invasive plants (BPA) of *C. spinifex* (*p* < 0.001) (Figure 1a–d). Notably, the all-grasses combination (P) showed the strongest inhibitory effect on the growth of *C. spinifexs*: under low-nitrogen conditions, its total biomass and seed biomass were significantly lower than those of other combinations; even under high-nitrogen treatment, the total biomass and seed biomass of *C. spinifex* in the P combination were still significantly lower than those in the combinations containing legumes (PL, PLA, LF) (Figure 1a,b). Root surface area generally increased under high-nitrogen conditions, but the increase was the smallest in the P combination, further indicating that this combination had a stronger inhibitory effect on the root expansion of invasive plants (Figure 1c). The BPA of invasive plants showed significant differences among different plant combinations: the BPA of the all-grasses combination (P) was the lowest under both low- and high-nitrogen conditions, indicating its strongest resistance to invasion; while the BPA of the legume and forbs combination (LF) was the highest, showing its weakest inhibitory effect on invasion (Figure 1d). 

The results of two-way ANOVA (Table S1) showed that local plant species composition had a highly significant effect on the number of tillers, seed number, root length, seed biomass, total biomass and BPA of *C. spinifex* (*p* < 0.001). Especially in key growth indicators such as number of tillers, seed number and total biomass, there were highly significant differences between the P combination and other combinations, further confirming the systematic inhibitory effect of the all-grasses community on the growth of *C. spinifex*. In addition to the significant effects of nitrogen addition on the above indicators, there were also significant interactions between nitrogen addition and species composition in number of tillers, seed number, seed biomass and total biomass (*p* < 0.05), indicating that the P combination could maintain a strong inhibitory capacity against invasion under both low and high-nitrogen conditions, and its resistance mechanism was relatively less affected by nitrogen input.

Further analysis of the growth traits of *C. spinifex* showed that nitrogen addition significantly increased its plant height, number of tillers, seed biomass, root length, root–shoot ratio and root volume (Figure S1a–f). However, in the P combination, the increase in plant height, number of tillers and seed biomass due to nitrogen addition was significantly lower than that in other combinations, especially in number of tillers and seed biomass, the values of the P combination under high-nitrogen conditions were still significantly lower than those of the PL and PLA combinations (Figure S1b,c). This indicates that the all-grasses community can not only effectively inhibit the basic growth of *C. spinifex*, but also significantly limit its reproductive investment under increased resource conditions.

The composition of local plant species has a significant impact on the nutrient status of *C. spinifex* and its rhizosphere soil, especially on the total phosphorus content of plants and the total nitrogen and NO_3_-N of soil (*p* < 0.001, *p* = 0.025) (Table 1). Under low-nitrogen (CK) conditions, the total nitrogen content of soil in the PL combination (*Poaceae* + *Fabaceae*) (1.266 ± 0.008 mg/kg) was significantly higher than that in the P, PLA and LF combinations, indicating that *Fabaceae* plants can effectively increase the nitrogen pool level in the rhizosphere soil even in a low-nitrogen environment.

Nitrogen addition treatment significantly promoted the accumulation of nitrogen and phosphorus nutrients in plants and soil (*p* < 0.001) (Table 1). Nitrogen addition increased the total nitrogen content of *C. spinifex* plants by 284–515% and the total phosphorus content by 329–879%. Under nitrogen addition conditions, the total nitrogen content of soil in all treatments containing *Fabaceae* plants (PL, PLA, LF) was significantly higher than that in the pure *Poaceae* combination(P).

There was a highly significant interaction between the two treatment factors (species composition and nitrogen addition) on the total phosphorus content of plants and the total nitrogen content of soil (*p* < 0.001), indicating that the composition of local plant communities significantly regulated the nutrient response pattern of the system to nitrogen input (Table 1). Specifically, in the PL combination, nitrogen addition significantly reduced the nitrate nitrogen content of soil by 38% (*p* = 0.007); while in the PLA combination, the total phosphorus content of soil (0.463 ± 0.110 mg/kg) was significantly higher than that in other treatments.

In summary, under low-nitrogen conditions, the total nitrogen content of soil in the PL combination was significantly higher than that in other combinations; while under high-nitrogen conditions, the total nitrogen content of soil in all combinations containing *Fabaceae* plants (PL, PLA, LF) was significantly higher than that in the P combination. In addition, the total phosphorus content of soil in the PLA combination under high-nitrogen was also significantly higher than that in other combinations. These results suggest that the presence of *Fabaceae* plants and their interaction with nitrogen addition are key factors in regulating the availability of nitrogen and phosphorus nutrients in the rhizosphere soil.

### 2.2. Effects of Nitrogen Addition and Species Combination on Soil Microorganisms in C. spinifex

#### 2.2.1. Analysis of Alpha Diversity in Soil Bacterial and Fungal Communities

**Figure 2:**
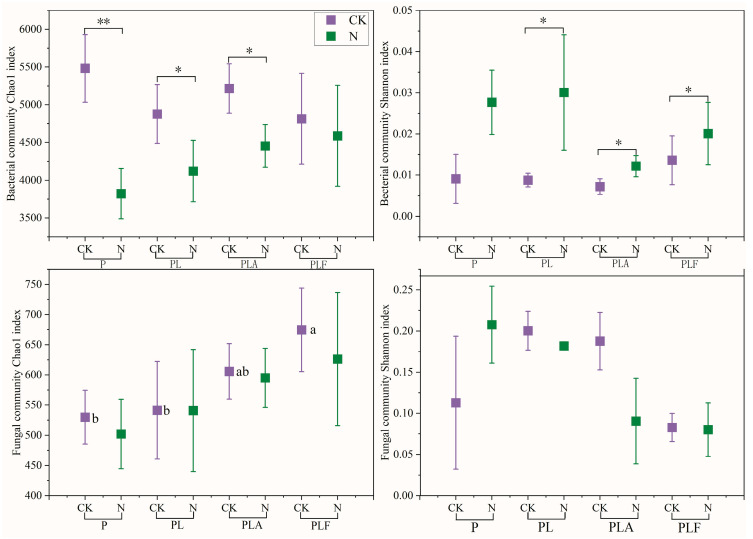
Diversity and richness indexes of bacterial and fungal community at the OTU level among different treatments. Different lowercase letters indicate significant differences among different species combinations; the presence of * (*p* < 0.05) and ** (*p* < 0.01) indicates significant differences among different N environments within the same species combination.

The results of the Alpha diversity of the bacterial community (Figure 2) showed that nitrogen addition (N) treatment significantly reduced the Chao1 index of the bacterial community, but increased the Shannon index. This trend was consistent across different plant functional group combinations, indicating that exogenous nitrogen input exerted a certain environmental screening pressure on the bacterial community, leading to a simplification of the community structure. The Alpha diversity of the fungal community, however, exhibited a different response pattern (Figure 2). The richness (Chao1 index) and diversity (Shannon index) of fungi did not show the same clearly opposite changes across different treatments and combinations as observed in the bacterial community. Overall, this Alpha diversity analysis revealed significant differences in the responses of soil microbial communities to treatments: the species richness and diversity of the bacterial community were highly sensitive to exogenous nitrogen input, while the diversity structure of the fungal community demonstrated greater stability and resistance.

#### 2.2.2. Analysis Results of Bacterial and Fungal Community Composition

**Figure 3:**
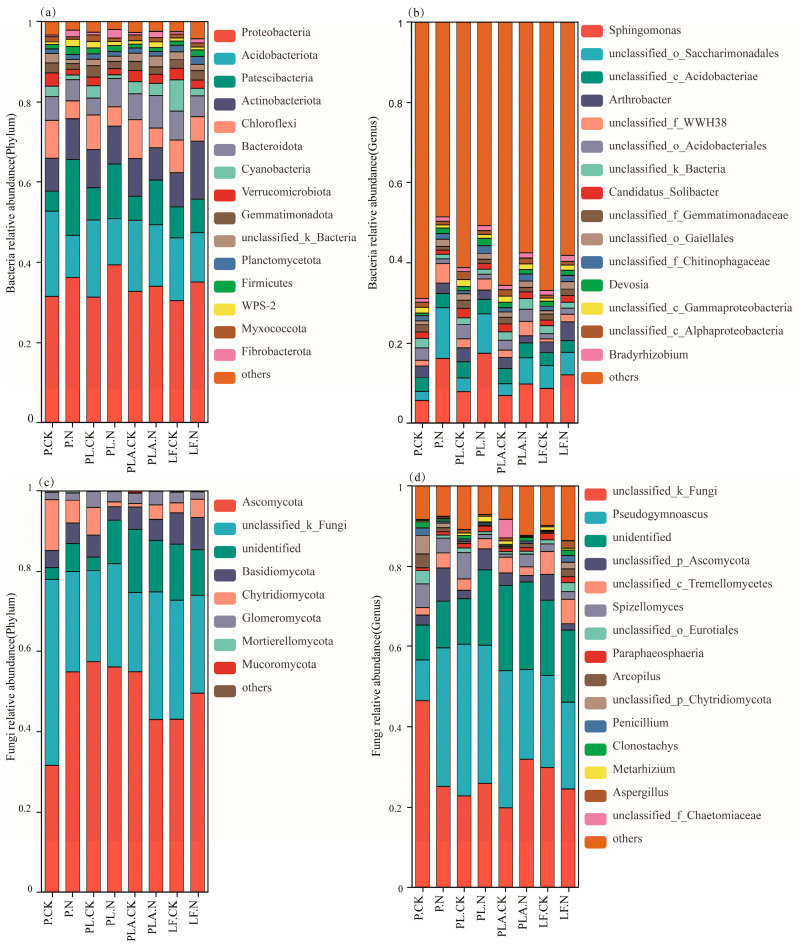
Relative abundances of bacterial communities at the phylum level (**a**), genus level (**b**), and fungal communities at the phylum level (**c**) and genus level (**d**) in different samples.

The soil sample sequences were analyzed, and the species with the top 15% abundance at the phylum and genus levels were selected to draw the cumulative bar chart of species relative abundance. “Others” represented the species with a relatively low proportion of less than 1%, and the species without biological annotation were classified as “unidentified”. At the phylum classification level, the composition of the bacterial community underwent systematic changes (Supplementary Table S2). In all treatments, Proteobacteria and Actinobacteriota were the dominant phyla, and their combined relative abundance accounted for more than 65% of the total bacterial community (Figure 3a). Nitrogen addition significantly affected the relative abundance of multiple bacterial phyla. Among them, the relative abundance of Acidobacteriota decreased significantly in all plant combinations due to nitrogen addition (*p* < 0.001), for example, from 0.218 to 0.107 in the P combination. In contrast, the relative abundance of Patescibacteria generally increased significantly after nitrogen addition (*p* = 0.004). Notably, there was a highly significant interaction between local species composition and nitrogen addition on the relative abundance of Actinobacteriota (*p* = 0.002). In the LF combination (legumes + forbs), nitrogen addition led to a sharp increase in the abundance of Actinobacteriota from 0.087 to 0.147, which was significantly higher than that in other plant combinations (Supplementary Table S2).

At the genus classification level, the responses of key bacterial groups to treatment combinations were further refined (Supplementary Table S3). The relative abundance of *Sphingomonas* was significantly regulated by the interaction between species composition and nitrogen addition (*p* < 0.001). Under nitrogen addition conditions, the abundance of this genus in the P and PL combinations was significantly higher than that in their respective controls. The abundance of *Devosia* was also significantly affected by the interaction of the two factors (*p* = 0.002), and it increased significantly in the P and PL combinations due to nitrogen addition. Additionally, the relative abundance of *Bradyrhizobium*, a genus related to nitrogen fixation, showed an overall upward trend after nitrogen addition (*p* = 0.008).

Compared with the bacterial community, the composition structure of the fungal community at the phylum level exhibited higher stability (Supplementary Table S4). Ascomycota and Basidiomycota were the absolute dominant phyla, and their relative abundances did not show significant changes among different treatments. However, the relative abundance of Chytridiomycota was significantly affected by local species composition (*p* = 0.006), and its abundance in the P combination control treatment (0.254) was significantly higher than that in the PL, PLA, and LF combinations. There was no statistical difference in the relative abundance of Glomeromycota among the treatments.

The analysis of fungal genus-level data revealed more functionally indicative changes in groups (Supplementary Table S5). The relative abundance of *Spizellomyces* was significantly affected by species composition (*p* = 0.008), and it was the highest in the P combination control. The abundance of *Arcopilus* was also significantly affected by species composition (*p* = 0.004) and interacted with nitrogen addition (*p* = 0.012), and its abundance in the P combination control was significantly higher than that in other combinations.

**Figure 4:**
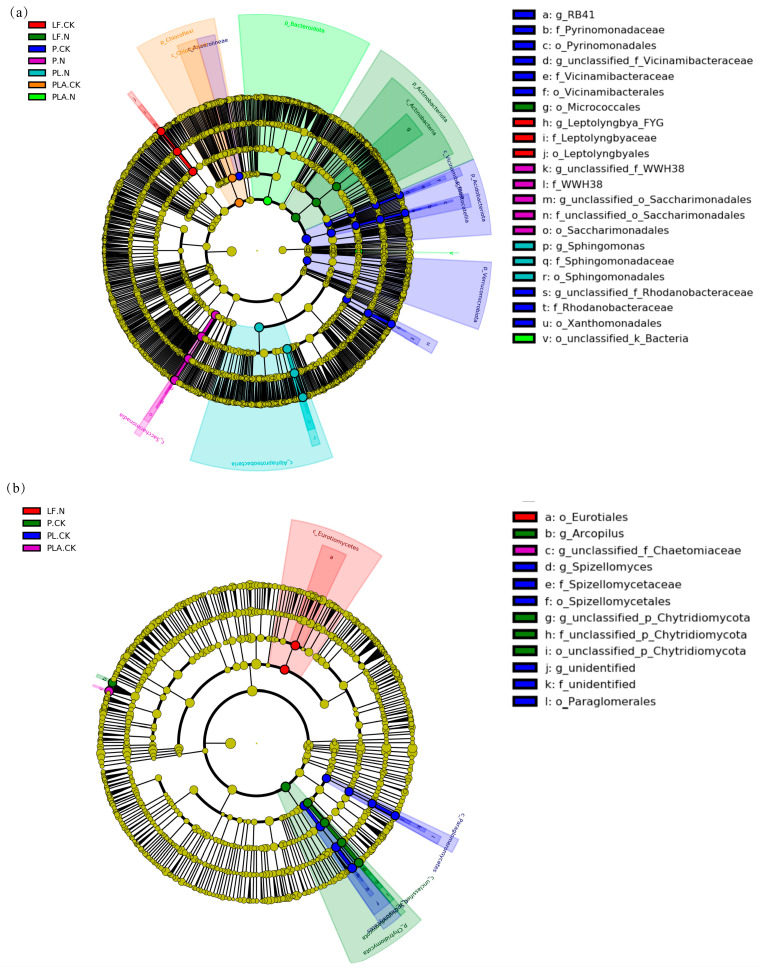
LEfSe multi-level species hierarchical tree of soil bacteria (**a**) and fungi (**b**).

Through LEfSe analysis (Figure 4), a total of 12 fungal and 20 bacterial taxa were identified as significantly enriched in the legume-containing combinations (especially PL and PLA). Among the bacteria, these taxa mainly belonged to Proteobacteria, Cyanobacteria, and Acidobacteriota, including *Rhizobium*, which is known to be related to nitrogen fixation, and RB41 in the *Pyrinomonadaceae* family. In fungi, Chaetomiaceae and Glomeromycota showed significant responses in specific treatments. These results suggest that Proteobacteria, Cyanobacteria, and nitrogen-fixing potential fungal taxa may play important roles in nitrogen acquisition and transformation in the rhizosphere of *C*. *spinifex* , especially under the presence of legumes, where the enrichment of these taxa may further enhance the rhizosphere nitrogen cycling function.

### 2.3. The Synergistic Interaction Among Plant Traits, Soil and Microorganisms Affects the Invasion of C. spinifex

**Figure 5:**
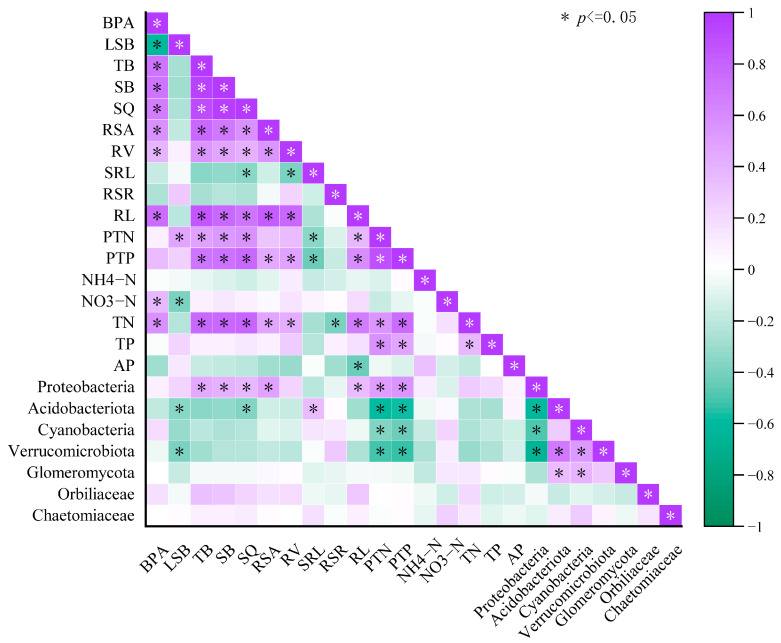
Heatmap of the Correlation between Plant Traits and Soil and Microbial Diversity. BPA: biomass proportion of invasive plants; LSB: total biomass of local community; TB: total biomass of invasive plants; SB: seed biomass; SQ: seed quantity; RSA: root surface area; RV: root volume; SRL:specific root length; RSR: root shoot ratio; RL: root length; PTN: total nitrogen content of plants; PTP: total phosphorus content of plants; NH_4_-N: soil ammonium nitrogen content; NO_3_-N: soil nitrate nitrogen content; TN: total nitrogen content of soil; TP: total phosphorus content of soil; AP: available phosphorus in soil. * symbol indicates a significant correlation (*p* < 0.05).

The species-specific bacteria obtained from the LEfSe analysis were screened, and four bacteria related to nitrogen fixation and three fungi were selected. Correlation analyses were conducted with plant traits, nutrients, and soil nutrients. The correlation heatmap analysis indicated that the growth and reproductive traits of *C*. *spinifex* (including above-ground biomass, root morphology indicators such as root surface area, root volume, root length, and seed yield) were significantly positively correlated with the biomass proportion of invasive plants (BPA) (Figure 5). The total biomass of local plants was significantly negatively correlated with BPA. Soil total nitrogen content was significantly positively correlated with plant nitrogen and phosphorus contents. The Proteobacteria was significantly positively correlated with the growth and reproductive traits and the nutrient content of *C. spinifex*, while the Cyanobacteria, Acidobacteriota, and Verrucomicrobiota showed significant negative correlations with the nitrogen and phosphorus contents of *C. spinifex*.

**Figure 6:**
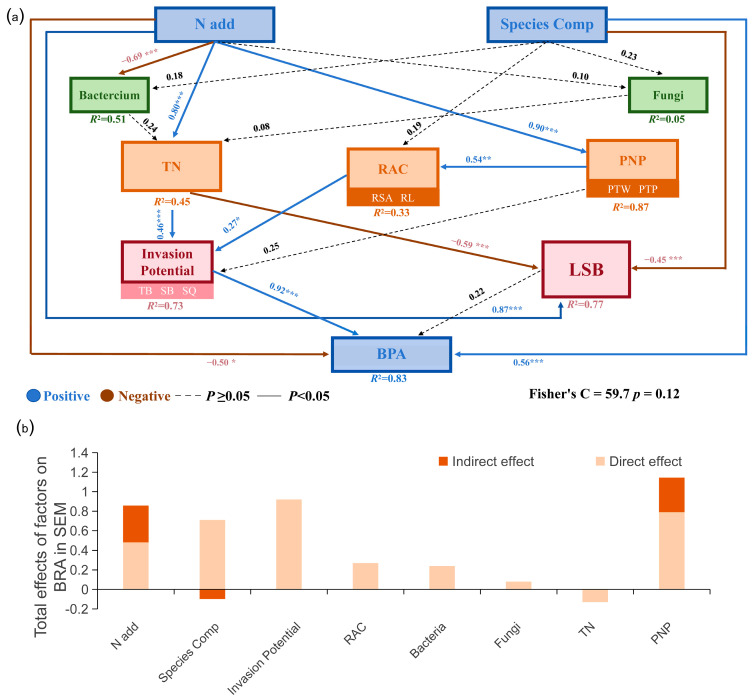
(**a**) Structural equation model of the effects of nitrogen addition and species composition on the invasion success rate of *C. spinifex*. (**b**) Total effects of each factor in the structural. The numbers beside the lines represent path coefficients. Solid lines indicate significant relationships, and dashed lines indicate insignificant ones. Asterisks indicate the significance level (* *p* < 0.05, ** *p* < 0.01, *** *p* < 0.001). “Invasion Potential” represents invasion potential (characterized by total biomass, seed biomass, and seed number), “RAC” represents resource acquisition capacity (root surface area, root length), “BPA” represents the biomass proportion of invasive plants, and “LSB” represents the biomass of local plants. The segmented structural equation model (SEM) explained 83% of the variance in the biomass proportion of invasive plants (BPA) (*R*^2^ = 0.83) and 73% of the variance in the invasion potential (*R*^2^ = 0.73).

Nitrogen addition was the core variable driving the invasion process (Figure 6a). It had a significant direct negative effect on the biomass proportion of invasive plants (BPA) (path coefficient = −0.50, *p* < 0.05), which might reflect the enhanced competition of the local community (especially the P combination) under high-nitrogen conditions. However, the promotion effect of nitrogen addition through multiple indirect paths was more crucial: it significantly directly increased the total soil nitrogen pool (path coefficient = 0.80, *p* < 0.001), and significantly inhibited the bacterial community structure (path coefficient = −0.69, *p* < 0.001). The increase in total soil nitrogen significantly enhanced the invasion potential of the *C*. *spinifex* (path coefficient = 0.46, *p* < 0.01) and inhibited the local plant biomass (LSB, path coefficient = −0.59, *p* < 0.001).

The influence of local species composition on the invasion process was mainly achieved through indirect paths(Figure 6a). It had a significant direct negative effect on the local plant biomass (LSB) (path coefficient = −0.45, *p* < 0.001), indicating that different species configurations directly affect the biomass accumulation of the community. At the same time, species composition had a significant direct positive effect on BPA (path coefficient = 0.56, *p* < 0.001), which might be related to specific combinations (such as legumes) directly promoting invasion. By suppressing LSB, species composition indirectly weakened the community’s biological resistance (path coefficient = 0.22, *p* = 0.21).

Plant resource acquisition ability (RAC) is the key plant trait connecting soil nutrients and invasion success. The plant nitrogen and phosphorus content (PNP) has a highly significant positive promoting effect on RAC (path coefficient = 0.54, *p* < 0.01), and the enhancement of RAC significantly increased the invasion potential (path coefficient = 0.27, *p* < 0.05) (Figure 6a), indicating that the plasticity of root morphology is an important mechanism for the competitive advantage of the *C*. *spinifex* in obtaining resources.

Nitrogen addition had a highly significant inhibitory effect on the bacterial community structure (path coefficient = −0.69, *p* < 0.001), indicating that nitrogen input reshaped the rhizosphere bacterial community(Figure 6a). The path coefficient from bacterial community to invasion potential was negative (−0.24), but this effect was not statistically significant (p = 0.24). Therefore, no firm conclusion can be drawn about the direct role of bacterial communities in invasion resistance from this model alone. The fungal community (Fungi_PCA) had an extremely low explanatory rate in this model (*R*^2^ = 0.05), indicating that its role in driving the overall invasion outcome was relatively limited.

Total effect analysis of each factor on overall invasion success further elucidated their relative contributions within the conceptual driving framework (Figure 6b). Plant nitrogen and phosphorus content (PNP) exhibited the strongest total effect on invasion success (1.14), underscoring that the intrinsic nutrient status of the invasive species is the most critical proximate determinant of invasion success. Nitrogen addition followed closely, with a total effect of 0.86—confirming its role as a central abiotic driver. This net positive effect arises from the offsetting combination of a direct negative pathway (−0.50) and stronger indirect positive pathways, primarily mediated through increased soil total nitrogen (+0.80) and subsequent suppression of native plant biomass (−0.59). Local plant species composition exerted a substantial total effect of 0.61, reflecting its multifaceted influence on invasion outcomes via interconnected aboveground and belowground mechanisms.

## 3. Discussion

### 3.1. Functionally Similar Local Communities Jointly Suppress Invasions Through Phylogenetic Association, Resource Competition and Microbial Regulation

This study demonstrates that local communities composed exclusively of Poaceae species (the P combination) exhibit the strongest resistance to the invasion of *C. spinifex* This finding not only provides support for the ‘limiting similarity hypothesis’ [23] but also aligns with the niche overlap implied by their relatively close phylogenetic distance (PD = 189) [17]. Phylogenetically related species tend to share greater similarity in resource-use strategies and functional traits [15]. Consequently, native *Poaceae* species in the P combination likely exhibit high overlap with *C. spinifex* in key traits such as root architecture and nutrient uptake, thereby driving direct competitive exclusion within the limited soil nitrogen pool—consistent with observations from grassland ecosystem studies by Byun [4].

Furthermore, this study provides evidence from belowground processes: under low-nitrogen conditions, rhizosphere soils of the P combination maintained a relatively high relative abundance of the phylum Acidobacteria. Acidobacteria are typically associated with oligotrophic environments characterized by slow resource turnover [24]. Structural equation modeling further revealed that communities dominated by phylogenetically similar species may enhance the system’s capacity for competitive nutrient interception and retention by shaping microbial communities that drive conservative resource cycling. This forms a resistance pathway linking aboveground phylogenetic associations with belowground microbial functional synergies, consistent with the plant–soil feedback theory proposed by Kulmatiski [25].

In contrast, combinations with greater phylogenetic distances (e.g., the LF assemblage, PD = 608) may exhibit interspecific trait divergence that fosters niche complementarity, thereby reducing overall competitive pressure on invasive species [3]. This suggests that phylogenetic distance can serve as a useful predictor of local community invasion resistance [26]. In low-nitrogen environments, constructing local communities phylogenetically close to invasive species represents an effective biotic resistance strategy that integrates aboveground competition and belowground microbial regulation.

### 3.2. Nitrogen Addition Affects Competitive Patterns and Invasion

The effect of nitrogen addition on invasion outcome is inherently dualistic, yet quantitatively dominated by indirect positive pathways. Although a statistically significant direct negative path from nitrogen addition to BPA was detected (−0.50, *p* < 0.05)—potentially attributable to transient competitive enhancement of certain native species under elevated nitrogen availability [12,27]—this direct effect must be interpreted within the broader causal network rather than in isolation. Crucially, nitrogen addition simultaneously triggers robust indirect positive effects: it significantly elevates soil total nitrogen pools (path coefficient = 0.80), which in turn directly enhances invasion potential (+0.46) and suppresses native plant biomass (−0.59) (Figure 6a). Summing the direct negative effect with all mediated indirect effects yields a net total effect of +0.86 on BPA (Figure 6b), confirming that the cumulative strength of indirect pathways exceeds that of the direct pathway. This net positive influence is consistent with the resource fluctuation hypothesis [28], and supports theoretical and empirical predictions that anthropogenic nitrogen enrichment increases plant invasion risk at regional to global scales [8,29]. Nitrogen addition significantly and directly increased the total soil nitrogen pool (path coefficient = 0.80, *p* < 0.001), thereby directly promoting the invasion potential of *C*. *spinifex*. (path coefficient = 0.46, *p* < 0.01). At the same time, nitrogen addition significantly suppressed the biomass of native plants (path coefficient = −0.59, *p* < 0.001), weakening the biological resistance of the community.

Plant nitrogen and phosphorus content (PNP) was an important driver of the resource acquisition ability of invasive plants in this study. The model revealed that plant nitrogen and phosphorus content significantly promoted the resource acquisition ability (RAC) (path coefficient = 0.54, *p* < 0.01), and thereby indirectly enhanced the invasion potential (path coefficient = 0.27, *p* < 0.05). This result is consistent with the conclusion of that *C*. *spinifex* has a strong ability to absorb and utilize nitrogen and phosphorus, indicating that the accumulation of nitrogen and phosphorus in plants is the internal basis for maintaining their competitive advantage [29]. This coherent “nitrogen input—soil nutrient increase—plant nitrogen and phosphorus accumulation—enhanced resource capture—increased invasion potential” path quantifies the specific operation mechanism of the resource fluctuation hypothesis [29] in this system and highlights that the functional responses of plants (such as increased root surface area and root length) are the direct manifestations of obtaining competitive advantages [30].

The response and role of the microbial community in this process are important but not direct regulatory factors. Nitrogen addition significantly inhibited the overall structure of the bacterial community (path coefficient = −0.69, *p* < 0.001), specifically manifested as a decrease in community richness and a reduction in the relative abundance of oligotrophic groups such as Acidobacteria and an increase in groups such as Patescibacteria. This shift towards a “eutrophic” microbial community composition is consistent with the characteristics of a high-nitrogen soil environment [31,32]. Although the direct effects of bacteria on TN and invasion potential in the SEM were not significant, their community structure is closely related to soil nutrient conditions, indicating that the changes in the microbial community are more a response and a co-factor of resource environment changes, and they may further enhance the availability of resources for invasive plants by accelerating nutrient turnover [33].

It is worth noting that the total effect of plant nitrogen and phosphorus content (PNP) is as high as 1.14, exceeding that of nitrogen addition itself, indicating that the nutrient accumulation status of invasive plants themselves plays a decisive role in their successful invasion. The nitrogen and phosphorus content in plants is a key indicator reflecting their nutrient absorption and utilization capabilities. High nitrogen and phosphorus content implies that plants have greater growth potential and reproductive output, thereby gaining an advantage in competition [13,30]. Model results further confirm that PNP indirectly enhances the invasion potential by promoting the root system’s resource acquisition ability, highlighting the central role of the plant’s internal nutrient status in resource capture. This finding suggests that, under the background of nitrogen deposition, in addition to focusing on soil nutrient availability, attention should also be paid to the strategies of invasive plants for nutrient absorption, transport, and utilization. The coupling of nitrogen and phosphorus within plants may be an important source of their competitive advantage. Previous studies have shown that the nitrogen-to-phosphorus ratio in plants can affect their growth rate and resource allocation patterns, thereby regulating their responses to environmental changes [34]. Therefore, future research should further reveal the intrinsic connections between plant nitrogen and phosphorus content and root physiology, as well as microbial interactions, to gain a more comprehensive understanding of the invasion mechanism.

### 3.3. The Shifting Role of Leguminous Plants in High-Nitrogen Environments

Under nitrogen addition conditions, the combination containing leguminous plants (PL) transformed from a competitor to a significant “synergist” promoting the invasion of *C. spinifex* (Figure 2 and Figure S1). Leguminous plants not only directly increased the total nitrogen and total phosphorus content in the rhizosphere soil through symbiotic nitrogen fixation (Table 2), but more importantly, significantly altered the soil microbial community. LEfSe analysis, in conjunction with community composition data, indicated that the PL combination enriched a series of related microbial groups centered around Proteobacteria at high nitrogen levels (Figure 4a and Figure 5). At the phylum level, the relative abundance of Proteobacteria increased the most; at the genus level, Sphingomonas and Bradyrhizobium showed an increasing trend. Among them, Proteobacteria, as one of the key groups with the greatest metabolic diversity and ecological functions in soil bacteria, is widely involved in the cycling of multiple elements such as carbon, nitrogen, and sulfur, as well as in symbiotic or growth-promoting processes with plants [35]. Sphingomonas is widely recognized for its potential in organic matter degradation and plant growth promotion [36], while Bradyrhizobium is a key symbiotic nitrogen-fixing bacterium with rich nitrogen-fixing gene resources in its genome [37]. These microbial groups are typically associated with active carbon and nitrogen cycling and nutrient-rich environments.

Although the direct effect of species composition on bacterial community structure was not significant (path coefficient = 0.18, *p* = 0.17), it indirectly influenced the functional composition of the microbial community by shaping a high-nutrient rhizosphere microenvironment (such as increasing soil total nitrogen and total phosphorus). As an environmental response hub (*R*^2^ = 0.51), the structural changes to the bacterial community were closely coupled with soil nutrients, jointly mediating the role transformation of leguminous plants from “competitor” to “synergist”. This fundamental role transformation warns that when introducing leguminous plants under nitrogen deposition, a cautious assessment is needed, as their originally beneficial nitrogen-fixing function may, through altering the structure and function of underground microbial communities, have unexpected promoting effects on specific invasive plants [10,38]. Similar phenomena have also been reported in North American grassland studies, where leguminous plants in a nitrogen-rich environment may support the expansion of non-native grasses by altering the nitrogen cycle [39].

### 3.4. Microbial Feedback Is the Core That Links Aboveground Intervention with Underground Processes

Bacterial communities play a significant regulatory role. Although the SEM suggested a negative path from bacterial community to invasion potential, this effect was not statistically significant (*p* = 0.24). Therefore, we cannot conclude that bacterial communities directly drive invasion resistance based on this model. Instead, the observed shift in bacterial community composition under nitrogen addition (e.g., decreased Acidobacteria, increased Patescibacteria) is consistent with a transition toward eutrophic conditions [32,33], but the functional consequences for invasion remain to be experimentally validated. The stronger resistance of the grass-only *Poaceae* community (P combination) may instead be explained by direct resource competition and phylogenetic relatedness, as discussed in Section 3.1, rather than by bacterial mediation.

Fungal communities, however, contributed minimally in this study. The variation explained by fungal communities in the model was extremely low (*R*^2^ = 0.05), indicating their relatively weak role in driving the overall invasion outcome, possibly due to their slower response to environmental changes or functional redundancy [40]. Nevertheless, certain specific fungal groups (such as arbuscular mycorrhizal fungi) may influence plant competition patterns at a local scale by promoting phosphorus absorption [40], which needs to be explored through more detailed functional validation in future studies. However, certain specific fungal groups (such as arbuscular mycorrhizal fungi) may influence plant competition patterns at a local scale by promoting phosphorus uptake [41], which requires more detailed functional validation in future research.

Nitrogen addition weakened the biological resistance of the system by reshaping the microbial community. Models confirmed that nitrogen addition had a strong inhibitory effect on the bacterial community structure (path coefficient = −0.69, *p* < 0.001), which corresponded to the observed decline in bacterial richness and the decrease in the abundance of Acidobacteria and the increase in nutrient-rich groups in the experiments [7]. Particularly in combinations containing leguminous plants, nitrogen addition, in synergy with leguminous plants, shaped a rhizosphere microenvironment centered around Proteobacteria and other groups, enriching nitrogen-fixing and nutrient cycling-related genera, thereby reversing the competitive effect of the phylogenetic background to a nutrient promoting effect, and ultimately mediating the transformation of leguminous plants from competitors to invasive enhancers.

Therefore, microbial feedback is the core bridge connecting the phylogenetic background of aboveground plant communities, resource input, and underground ecological processes. The ultimate outcome of invasion not only depends on the functional group identity and phylogenetic distance aboveground but also on how these aboveground factors influence the distribution and flow of resources between competing parties by regulating the structure and function of underground microbial communities [42]. In the total effect analysis, the total effect of the local plant species combination reached 0.61, fully confirming the profound impact of aboveground community composition on invasion outcomes through underground feedback.

### 3.5. Future Research Directions and Broader Implications

Several important questions remain for future investigation. First, while our greenhouse experiment provides mechanistic insights under controlled conditions, field validation is urgently needed. Natural environments exhibit spatial heterogeneity in soil properties and temporal variability in nitrogen deposition that may modulate the interactions observed here. Long-term field studies tracking invasion dynamics across nitrogen deposition gradients would test the robustness of our findings. The generality of the legume-switch phenomenon—where leguminous plants transition from competitors to invasion facilitators under elevated nitrogen—remains unknown. Comparative studies across different invasive species with varying functional traits, as well as across different native species assemblages and ecosystem types (grasslands, forests, wetlands), would determine whether this finding is specific to *C. spinifex* or represents a broader ecological principle. Although fungal communities explained little variation in our SEM (*R*^2^ = 0.05), specific fungal functional groups such as arbuscular mycorrhizal fungi (AMF) may still play important roles at finer scales [43]. Future studies employing amplicon sequencing with higher taxonomic resolution, coupled with functional assays (e.g., AMF colonization rates, phosphorus uptake measurements), could reveal cryptic fungal effects.

From a management perspective, our results suggest actionable strategies for regions experiencing elevated nitrogen deposition. Maintaining or restoring phylogenetically similar grass communities (e.g., *Poaceae*-dominated combination) may enhance biotic resistance against *C. spinifex* and related invasive grasses. Conversely, introducing leguminous species in restoration projects—particularly in nitrogen-enriched environments—should be approached with caution, as their nitrogen-fixing function may inadvertently facilitate invasion. Finally, monitoring soil microbial community structure, especially the relative abundance of oligotrophic versus copiotrophic taxa, could serve as an early indicator of invasion risk.

## 4. Materials and Methods

### 4.1. Experimental Design

The study adopted a two-factor completely crossed design. The two factors were: (1) local plant species composition, with 4 levels; (2) nitrogen addition, with 2 levels (no nitrogen addition and nitrogen addition). Each treatment combination was repeated 4 times, resulting in a total of 32 experimental units (4 communities × 2 nitrogen levels × 4 replicates). The local plant species composition included 4 combinations, each with the same number of species but different functional group compositions (Table 2).

Nitrogen addition treatments: No nitrogen addition treatment (CK): The in situ soil collected from the invaded area was used, with a total nitrogen content of 0.25 g/kg. Nitrogen addition treatment (N): On the basis of the no nitrogen addition treatment, nitrogen was added by applying 150 mg/kg of analytical grade NH_4_NO_3_ (≥99% purity, dissolved in distilled water) to the soil This addition amount was based on the predicted high nitrogen deposition flux in northern China [43]. To reduce the intense impact of a single addition on the soil environment and simulate the continuous deposition process, nitrogen was applied in three equal amounts at two-week intervals.

### 4.2. Plant Materials and Cultivation Management

The experimental plants included the invasive species *C. spinifex* and 13 native plant species from its invaded area (Zhangwu County, Liaoning Province) (Table 2). All treatments were conducted in circular plastic pots (inner diameter × height: 30 cm × 25 cm), each filled with 13.5 kg of in situ soil collected from the *C. spinifex* invaded area in Zhangwu, Liaoning. The soil was taken from a sample point (at a depth of 0–20 cm) and was thoroughly mixed to form a soil sample. The use of in situ soil aimed to preserve the integrity of the native soil microbial community (including pathogens, arbuscular mycorrhizal fungi, etc.) as much as possible [44].

Seed treatment and seedling cultivation: On 7 June 2024, all plant seeds were disinfected and then sown. The seeds were soaked in a 2% NaClO solution for 10 min, followed by three rinses with deionized water to remove residual disinfectant. After the surface water was absorbed with disinfected filter paper, the seeds were immediately sown in 128-cell seedling trays filled with seedling substrate and placed in a greenhouse for cultivation. Greenhouse conditions: day/night temperature 25/22 °C, natural light. For each type of plant, 10–15 seeds will be sown in each 128-cell culture dish unit. The germination status will be monitored every day. The average germination time for different species ranges from 5 to 8 days, and the overall germination success rate averages 87% (ranging from 78% for the species of *Chenopodium acuminatum* to 95% for the species of *Setaria viridis*). After 20 days of cultivation, the young plants that have grown uniformly and healthily will be transplanted into flower pots.

**Figure 7:**
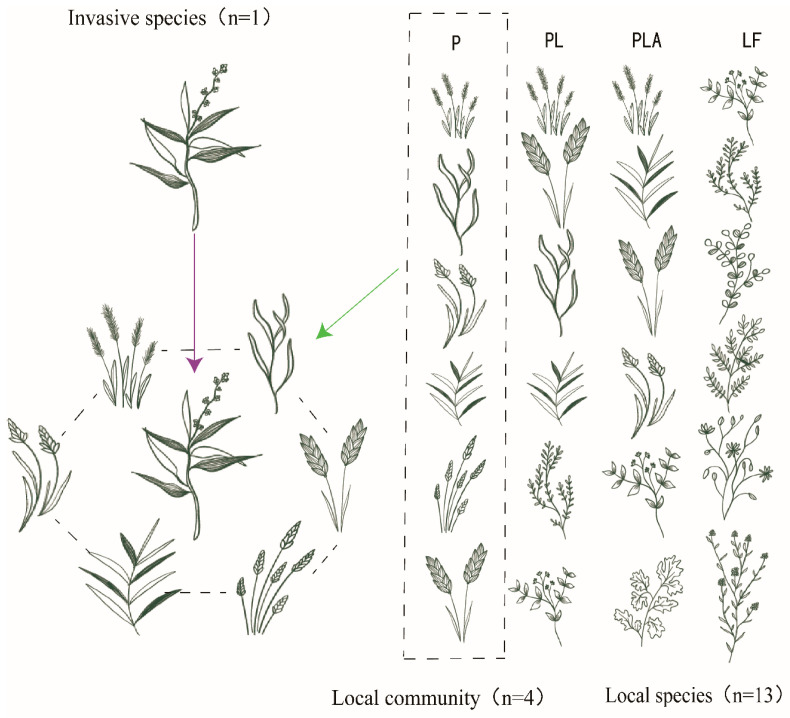
The planting positions of *C. spinifex* and native plants in the flowerpots.

Transplanting and planting: After 20 days of cultivation, healthy seedlings of the same size within the same species were selected for transplanting. The planting layout in each pot was as shown in Figure 7: one *C. spinifex* seedling was transplanted at the center of the pot, and six native plant seedlings were transplanted at equal intervals around it in a hexagonal pattern.

Cultivation management: After transplanting, all seedlings were watered adequately to help them acclimatize. Throughout the experiment, no nutrient solution was applied to highlight the main effect of nitrogen addition. Soil moisture in the pots was managed by weighing, and distilled water was added to maintain soil moisture content at 60–70% of field capacity. All potted plants were randomly placed in the greenhouse at the Bai Cao Yuan Base of Shenyang Agricultural University (41°50′ N, 123°34′ E).

### 4.3. Indicator Determination and Methods

On 12 September 2024, plant and soil samples were collected for the analysis of plant growth performance, nitrogen and phosphorus content, soil nutrient content, and microbial community structure, among other relevant indicators.

Plant growth performance: After measuring the final plant height, tiller number, and seed quantity of each pot of *C. spinifex*, all plants in the pots were removed, and the aboveground and root parts of *C. spinifex* and native species were gently shaken apart. The roots were rinsed with distilled water to remove adhering soil. The roots of *C. spinifex* were immediately placed in a sealed bag containing 20 mL of distilled water for preservation. The root morphology parameters of *C. spinifex*, including total root length, root surface area, and root volume, were determined using a root scanning and analysis system (WinRHIZO, Regent Instruments Inc., Québec City, QC, Canada). The plants of *C. spinifex* and native species were dried at 65 °C to a constant weight and weighed on a 0.0001 g balance to measure the biomass of each organ. All plant samples were ground and dried for storage, to be used for subsequent chemical analysis.

Soil sample collection: Three sampling points were set up in a triangular distribution around each *C. spinifex* plant. Soil samples from the 0–10 cm surface layer at each sampling point were collected using a 3 cm diameter stainless steel soil corer sterilized with 75% ethanol. The soil samples were passed through a 2 mm sterile sieve, mixed evenly, and divided into two equal parts. One part was stored at −80 °C for DNA extraction and sequencing, and the other part was stored at 4 °C for soil nutrient content determination.

Biomass ratio of invasive species: The dominance of *C. spinifex* in the community was expressed by calculating its biomass ratio [45].

$$Biomass\;proportion\;of\;alien\;target\;biomass=\frac{C.\;spinifex\;biomass}{C.\;spinifex\;biomass+Total\;biomass\;of\;native\;community}$$

Analysis of nitrogen and phosphorus content in plants and soil: Total nitrogen was determined by the Kjeldahl method, and total phosphorus was measured by the molybdenum–antimony–antimony colorimetric method. Soil ammonium and nitrate nitrogen concentrations were determined using a fully automatic chemical analyzer (SmartChem 200, AMS Alliance, Rome, Italy).

Analysis of soil microbial community structure: Bacterial and fungal community structures were analyzed through 16S rRNA gene and ITS (internal transcribed spacer) gene amplicon sequencing, respectively. After extracting total DNA from the soil, Guangdong Meige Gene Technology Co., Ltd. was entrusted to perform subsequent library construction and sequencing by Guangdong Meige Gene Technology Co., Ltd. (Shenzhen, China). The company used universal primers to amplify the V3–V4 hypervariable region of the 16S rRNA gene and the ITS1 or ITS2 region of fungi, and strictly followed the standard procedures of the ALFA-SEQ DNA Library Prep Kit(Alfa Genetics, Guangzhou, China) to construct sequencing libraries. Subsequently, the fragment size and quality of the libraries were evaluated using the Qsep400 high-throughput nucleic acid analysis system (Bioptic Inc., New Taipei City, Taiwan; distributed by Hangzhou Houze Biotechnology Co., Ltd., Hangzhou, China), and the Qubit 4.0 (Thermo Fisher Scientific, Waltham, MA, USA) was used for precise quantification. Finally, the constructed amplicon libraries were sequenced on the Illumina NovaSeq platform (Illumina, Inc., San Diego, CA, USA) using PE250 (250-base paired-end) sequencing.

### 4.4. Statistical Analysis

To quantify the phylogenetic structure of local plant communities, this study constructed a phylogenetic tree based on the ITS sequences of species and calculated the phylogenetic distance using piecewise structural equation modeling (Piecewise SEM) for analysis. The piecewiseSEM package in R software (R Foundation for Statistical Computing, Vienna, Austria) was used for model estimation. Firstly, a phylogenetic tree including all experimental species was constructed by the maximum likelihood method; subsequently, the Faith phylogenetic diversity index (PD) of each local plant combination was calculated, which is the minimum total length of phylogenetic branches required to cover all species in the combination. This index was used to characterize the phylogenetic differences among different functional group combinations and served as an explanatory variable in subsequent correlation and model analyses [17]. This index (Faith’s PD) was used to characterize phylogenetic differences among functional group combinations [17,46].

Data analysis was conducted using SPSS 27.0. Firstly, normal distribution (Shapiro–Wilk test) and homogeneity of variance (Levene test) were tested for the data. A two-way ANOVA was used to test the main effects and interaction effects of nitrogen addition and species composition on the invasion-related indicators of *C. spinifex* (such as biomass, proportion of invasive plant biomass, etc.). If the interaction was significant, a post hoc multiple comparison was conducted using the Duncan method. For the effect of a single factor (such as only nitrogen addition) at different species composition levels, an independent sample *t*-test was used for analysis.

To deeply reveal the direct and indirect influence paths of multiple factors such as nitrogen addition, species composition, soil nutrients, and microbial communities on invasion success, this study using piecewise structural equation modeling (Piecewise SEM) for analysis. The piecewiseSEM package in R software (R Foundation for Statistical Computing, Vienna, Austria) was used for model estimation. The overall model fit was evaluated by Fisher’s C statistic (*p* > 0.05 indicates a good fit). The explanatory power of each endogenous variable was expressed by R^2^, and the predictive relevance was evaluated by calculating Q^2^ through 10-fold cross-validation. The significance of path coefficients was tested by *t*-test, and the effect size was measured by Cohen’s f^2^ (f^2^ ≥ 0.02, 0.15, 0.35 represent small, medium, and large effects, respectively). Indirect effects were tested by Bootstrap resampling (1000 times), and significance was judged by a 95% confidence interval. Multicollinearity was diagnosed by the variance icnflation factor (VIF < 5). Based on the research hypotheses, an initial path model was constructed. Through the significance test of path coefficients and multiple model revisions, a well-fitted structural equation model was finally obtained. The standardized path coefficient diagrams in the model results were drawn and beautified using Microsoft Excel 2021 (Microsoft Corp., Redmond, WA, USA) and Adobe Illustrator 2024 (Adobe Inc., San Jose, CA, USA) software.

## 5. Conclusions

This study reveals that the successful invasion of *C. spinifex* is determined by the dynamic interaction among resource availability, the phylogenetic background of the local community, and the underground microbial processes it regulates. The grass family community with a relatively close phylogenetic distance (PD = 189) exhibits the strongest resistance to invasion through resource competition and conservative microbial feedback. Nitrogen addition is the core driving factor (total effect 0.86), which promotes invasion by increasing the soil nitrogen pool, suppressing local biomass, and reshaping the bacterial community. Leguminous plants transform from competitors to “synergistic” facilitators of invasion under high-nitrogen conditions, altering the rhizosphere microenvironment by enriching rapid nitrogen cycling microorganisms such as Proteobacteria. Plant nitrogen and phosphorus content (PNP) is the most critical indicator for predicting invasion success (total effect 1.14), highlighting the central role of the nutrient status within plants. The study emphasizes that ecological management should integrate the coordinated regulation of the phylogenetic background of aboveground functional groups and underground microbial processes.

Management implications: In regions experiencing elevated nitrogen deposition, we recommend (1) maintaining or restoring phylogenetically similar grass communities (e.g., *Poaceae*-dominated assemblages) to enhance biotic resistance; (2) exercising caution when introducing leguminous species in restoration projects under high nitrogen availability, as their nitrogen-fixing function may inadvertently facilitate invasion; and (3) incorporating soil microbial community structure (particularly oligotrophic vs. copiotrophic taxa ratios) as a monitoring indicator in invasion risk assessments.

## Figures and Tables

**Table 1:** Significance analysis of the effects of different species combinations and nitrogen addition on nutrient elements of *C. spinifex* and soil nutrients.

Nutrient Indicators	Treatment Combinations	Low Nitrogen (CK)	High Nitrogen (N)
Total nitrogen content of plants (mg/kg)	P	1.640 ± 0.353 *	6.887 ± 2.055 *
	PL	2.870 ± 0.790 *	6.027 ± 1.394 *
	PLA	2.950 ± 2.081 *	7.043 ± 1.471 *
	LF	1.328 ±0.648 ***	5.099 ± 0.600 ***
Total phosphorus content of plants (mg/kg)	P	4.399 ± 2.322 b ***	24.656 ± 2.479 ***
	PL	14.480 ± 0.867 a *	35.799 ± 1.201 *
	PLA	6.372 ± 3.641 b ***	30.385 ± 1.938 ***
	LF	3.758 ± 1.137 b***	28.677 ± 4.503 ***
Total nitrogen content in soil (mg/kg)	P	0.273 ± 0.0144 b	0.832 ± 0.144 b
	PL	1.266 ± 0.008 a	1.366 ± 0.160 a
	PLA	0.223 ± 0.042 b ***	1.644 ± 0.077 a ***
	LF	0.161 ± 0.124 b **	1.524 ± 0.250 a **
Total phosphorus content in soil (mg/kg)	P	0.086 ± 0.100	0.311 ± 0.225 b
	PL	0.076 ± 0.172	0.108 ± 0.087 b
	PLA	0.104 ± 0.108 **	0.463 ± 0.110 a **
	LF	0.100 ± 0.105	0.215 ± 0.256 b
NO_3_-N (mg/kg)	P	6.251 ± 0.641 b	5.960 ± 1.478
	PL	12.714 ± 2.660 ab	6.398 ± 1.611
	PLA	7.283 ± 2.577 b	12.888 ± 3.947
	LF	16.839 ± 7.676 a	11.778 ± 2.931
NH_3_-N (mg/kg)	P	3.893 ± 2.167	3.225 ± 1.072
	PL	3.708 ± 2.112	5.103 ± 4.287
	PLA	2.532 ± 0.800	2.313 ± 1.482
	LF	2.846 ± 1.444	3.015 ± 0.044

Note: The data in the table are presented as mean ± standard error (n = 4). Different lowercase letters indicate significant differences among different species combinations; the presence of * (*p* < 0.05), ** (*p* < 0.01), and *** (*p* < 0.001) indicates significant differences among different N environments within the same species combination.

**Table 2:** Information on local species used in the experiment.

Species	Family	Life Cycle	Assigned Treatment Group			
			P	PL	PLA	LF
*Setaria viridis*	*Poaceae*	Annual	√	√	√	
*Agropyron cristatum*	*Poaceae*	Perennial	√	√	√	
*Leymus chinensis*	*Poaceae*	Perennial	√			
*Cleistogenes caespitosa*	*Poaceae*	Perennial	√	√	√	
*Digitaria sanguinalis*	*Poaceae*	Annual	√	√	√	
*Calamagrostis epigeios*	*Poaceae*	Perennial	√			
*Lespedeza davurica*	*Fabaceae*	Perennial		√	√	√
*Astragalus scaberrimus*	*Fabaceae*	Perennial		√		√
*Astragalus laxmannii*	*Fabaceae*	Perennial				√
*Medicago ruthenica*	*Fabaceae*	Perennial				√
*Artemisia gmelinii*	*Asteraceae*	Perennial			√	
*Chenopodium acuminatum*	*Amaranthaceae*	Annual				√
*Potentilla tanacetifolia*	*Rosaceae*	Perennial				√
PD			189	405	471	608

Note: “√” means that the species is included in this treatment group.

## Data Availability

The datasets used in the current study are available from the corresponding author on reasonable request.

## Supplementary Materials

The following supporting information can be downloaded at: https://www.mdpi.com/article/10.3390/plants15132016/s1, Figure S1: Effects of nitrogen addition and local plant species composition on the growth performance of *C. spinifex*; Figure S2: Phylogenetic tree of the 14 plant species used in the experiment; Table S1: Two-way ANOVA results for the effects of local plant species composition and nitrogen addition on the invasion indicators of *C. spinifex*; Table S2: Significant analysis of the composition of soil microbial bacterial communities at the phylum level under different species combinations and nitrogen addition; Table S3: Significant analysis of the composition of soil bacterial communities at the genus level under different species combinations and nitrogen addition; Table S4: Significant analysis of the composition of soil fungal communities at the phylum level under different species combinations and nitrogen additions; Table S5: Significant analysis of the composition of soil fungal communities at the genus level under different species combinations and nitrogen additions.
