# Supplementary material for: The Composition of Native Plant Species and Nitrogen Availability Jointly Influence the Invasion Success of Cenchrus spinifex
Source: Plants (Basel). 2026 Jun 29;15(13):2016. doi: 10.3390/plants15132016 (PMC13364187; doi:10.3390/plants15132016)
Supplement: Supplementary file 1 [file plants-15-02016-s001.zip › plants-4328746-supplementary.pdf]

Supplementary Materials

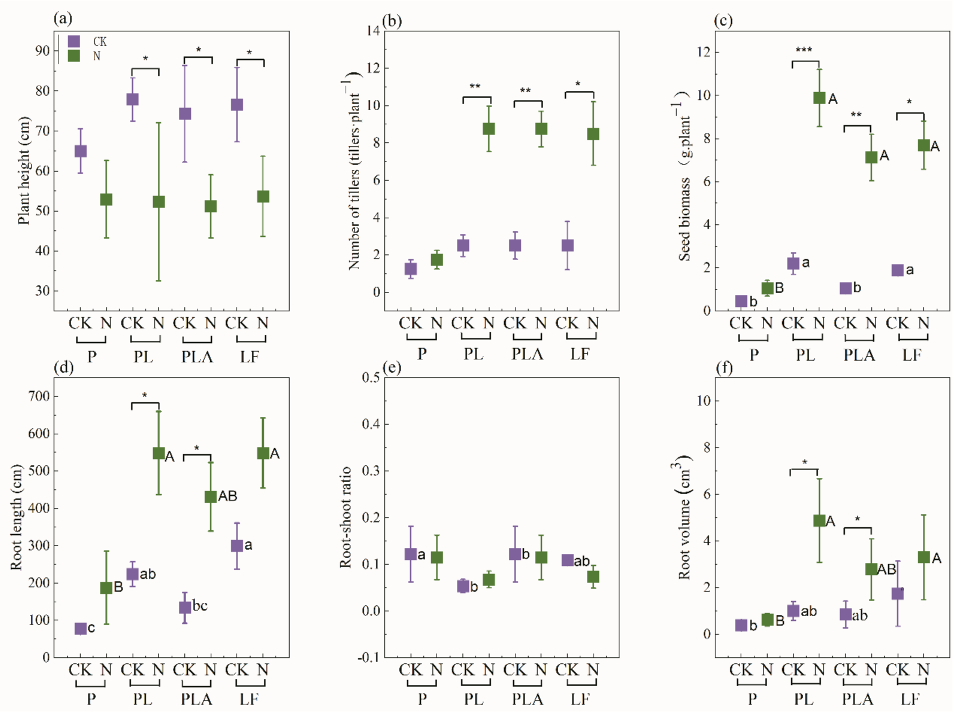

Figure S1: The effects of nitrogen addition and local plant species composition on the growth performance of *C. spinifex*. The figures in the graph represent (a) plant height, (b) number of tillers, (c) seed biomass, (d) root length, (e) root-shoot ratio, and (f) root volume of the *C. spinifex*. Different lowercase letters indicate significant differences among species combinations under the CK treatment ($p < 0.05$); different uppercase letters indicate significant differences among species combinations under the nitrogen application treatment ($p < 0.05$); and the presence of * ($p < 0.05$), ** ($p < 0.01$), and *** ($p < 0.001$) indicate the significance of differences in the same species combination under different N availabilities.

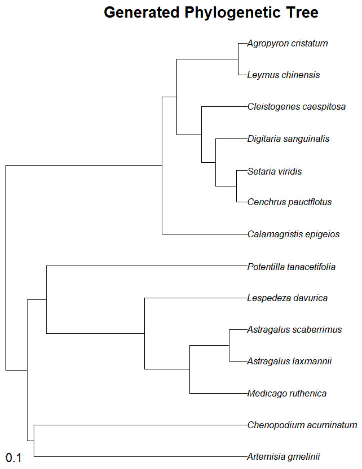

Figure S2: Phylogenetic tree of the species used in the experiment.

Table S1: Significance analysis of the effects of local plant species composition and nitrogen addition on the invasion indicators of *C. spinifex*.

Dependent variable	Species composition			N addition			Species composition × N addition		
	df	F	*p*	df	F	*p*	df	F	*p*
Plant height	3	0.576	0.064	1	29.816	**<0.001**	3	0.617	0.611
Number of tillers	3	9.889	**<0.001**	1	53.481	**<0.001**	3	4.765	**0.01**
Seed quantity	3	14.984	**<0.001**	1	108.046	**<0.001**	3	8.854	**<0.001**
Root length	3	5.758	**0.004**	1	21.452	**<0.001**	3	0.764	0.526
Root surface area	3	1.411	0.265	1	8.979	**0.006**	3	0.846	0.483
Root volume	3	0.282	0.838	1	10.557	**0.004**	3	0.029	0.993
Seed biomass	3	16.541	**<0.001**	1	82.549	**<0.001**	3	7.707	**<0.001**
Total biomass	3	14.667	**<0.001**	1	66.416	**<0.001**	3	5.972	**0.004**
Biomass ratio	3	0.421	**<0.001**	1	0.106	**<0.001**	3	0.022	**<0.001**

Table S2: Significant analysis of the composition of soil microbial bacterial communities at the phylum level under different species combinations and nitrogen addition.

Bacterial phylum	Treatment group	CK	N	Species combination	Nitrogen addition	Interaction	Significance
Proteobacteria	P	0.322±0.058	0.366±0.016	0.562	**0.001**	0.370	ns
	PL	0.318±0.013	0.400±0.053				*
	PLA	0.334±0.026	0.345±0.014				ns
	LF	0.310±0.042	0.356±0.041				ns
Acidobacteriota	P	0.218±0.030	0.107±0.016	0.352	**< 0.001**	**0.031**	***
	PL	0.193±0.034	0.119±0.031				*
	PLA	0.181±0.016	0.158±0.006				*
	LF	0.160±0.046	0.125±0.042				ns
Patescibacteria	P	0.050±0.047	0.190±0.087	0.531	**0.004**	0.153	*
	PL	0.080±0.037	0.137±0.066				ns
	PLA	0.062±0.017	0.115±0.032				*
	LF	0.080±0.077	0.085±0.058				ns
Actinobacteriota	P	0.084±0.008	0.103±0.013B	**0.021**	**0.018**	**0.002**	ns
	PL	0.098±0.027	0.096±0.025B				ns
	PLA	0.096±0.013	0.083±0.020B				ns
	LF	0.087±0.014	0.147±0.011A				***
Chloroflexi	P	0.098±0.022	0.045±0.016	0.928	**< 0.001**	0.293	**
	PL	0.090±0.023	0.050±0.012				*
	PLA	0.010±0.020	0.051±0.006				**
	LF	0.083±0.019	0.062±0.011				ns
Bacteroidota	P	0.061±0.019	0.054±0.036	0.263	0.590	0.084	ns
	PL	0.044±0.013	0.070±0.025				ns
	LF	0.066±0.007	0.083±0.022				ns

	LA	0.074±0.008	0.053±0.005				**
Cyanobacteria	P	0.027±0.011	0.013±0.010	0.088	**0.010**	0.115	ns
	PL	0.033±0.020	0.010±0.005				ns
	LF	0.030±0.012	0.031±0.031				ns
	LA	0.079±0.053	0.019±0.018				ns
Verrucomicrobiota	P	0.034±0.011	0.014±0.005	0.349	**0.002**	0.264	*
	PL	0.022±0.002	0.016±0.005				*
	PLA	0.028±0.003	0.024±0.004				ns
	LF	0.030±0.014	0.021±0.013				ns

Note: Capital letters indicate significant differences between different species combinations. Significance indicates the differences among different treatments of the same species combination; ns represents no statistical significance ($p \geq 0.05$); * $p < 0.05$; ** $p < 0.01$; *** $p < 0.001$, and the same applies below.

Table S3: Significant analysis of the composition of soil bacterial communities at the genus level under different species combinations and nitrogen addition

Bacterial genus	Treatment group	CK	N	Species combination	Nitrogen addition	Interaction	Significance
Sphingomonas	P	0.070±0.016	0.231± 0.053	0.153	0.070	**< 0.001**	*
	PL	0.101± 0.011	0.232± 0.062				**
	PLA	0.086± 0.015	0.133± 0.022				*
	LF	0.108 ±0.059	0.155± 0.095				ns
Arthrobacter	P	0.035±0.003	0.036±0.006AB	0.218	0.876	**0.036**	ns
	PL	0.046±0.027	0.031±0.004AB				ns
	PLA	0.035±0.012	0.025±0.012B				ns
	LF	0.032±0.007	0.058±0.023A				ns
Candidatus Solibacter	P	0.021±0.014	0.016±0.002	0.196	0.122	0.314	ns
	PL	0.030±0.003	0.019±0.007				**
	PLA	0.025±0.004	0.022±0.003				ns
	LF	0.018±0.004	0.021±0.009				ns
Devosia	P	0.008±0.002	0.019±0.003	**0.021**	**0.018**	**0.002**	***
	PL	0.015±0.002	0.024±0.006				**
	PLA	0.018±0.009	0.016±0.003				ns

	LF	0.016±0.005	0.017±0.006				ns
Bradyrhizobium	P	0.012±0.006	0.017±0.005	0.672	**0.008**	0.985	ns
	PL	0.013±0.003	0.017±0.004				ns
	PLA	0.015±0.004	0.019±0.002				ns
	LF	0.014±0.006	0.019±0.002				ns

Table S4: Significant analysis of the composition of soil fungal communities at the phylum level under different species combinations and nitrogen additions.

Fungal phylum	Treatment group	CK	N	Species combination	Nitrogen addition	Interaction	Significance
Ascomycota	P	0.572±0.155	0.701± 0.198	0.347	0.486	0.627	ns
	PL	0.733± 0.119	0.762± 0.124				ns
	PLA	0.686± 0.217	0.630± 0.069				ns
	LF	0.616±0.074	0.654± 0.083				ns
unidentified	P	0.059±0.033	0.098±0.119	0.106	0.526	0.608	ns
	PL	0.047±0.023	0.144±0.111				ns
	PLA	0.192±0.190	0.193±0.074				ns
	LF	0.193±0.119	0.152±0.074				ns
Basidiomycota	P	0.075±0.045	0.072±0.033	0.196	0.122	0.314	ns
	PL	0.077±0.039	0.043±0.026				ns
	PLA	0.071±0.035	0.075±0.040				ns
	LF	0.113±0.032	0.102±0.068				ns
Chytridiomycota	P	0.254±0.161a	0.095±0.115	**0.006**	0.167	0.062	ns
	PL	0.091±0.078b	0.015±0.018				ns
	PLA	0.011±0.007b	0.052±0.017				**
	LF	0.036±0.016b	0.067±0.073				ns
Glomeromycota	P	0.035±0.010	0.027±0.017	0.201	0.212	0.094	ns
	PL	0.052±0.018	0.034±0.011				ns
	PLA	0.033±0.011	0.049±0.014				ns
	LF	0.039±0.016	0.024±0.012				ns

Table S5: Significant analysis of the composition of soil fungal communities at the genus level under different species combinations and nitrogen additions.

Fungal genus	Treatment group	CK	N	Species combination	Nitrogen addition	Interaction	Significance
Pseudogymnoascus	P	0.230±0.104	0.516± 0.305	0.518	0.771	0.186	ns
	PL	0.497± 0.255	0.520± 0.245				ns
	PLA	0.491± 0.250	0.350± 0.141				ns

	LF	0.416±0.086	0.333± 0.116				ns
unidentified	P	0.218±0.038	0.199±0.169	0.324	0.621	0.724	ns
	PL	0.173±0.090	0.292.±0.218				ns
	PLA	0.315±0.219	0.315±0.132				ns
	LF	0.323±0.137	0.283±0.102				ns
Spizellomyces	P	0.154±0.072a	0.074±0.069	**0.008**	0.093	0.065	ns
	PL	0.094±0.083ab	0.014±0.018				ns
	PLA	0.011±0.007b	0.048±0.020				*
	LF	0.033±0.019b	0.032±0.045				ns
Paraphaeosphaeria	P	0.016±0.013	0.015±0.007	0.156	0.955	0.704	ns
	PL	0.015±0.004	0.022±0.021				ns
	PLA	0.013±0.007	0.014±0.004				ns
	LF	0.030±0.012	0.023±0.013				ns
Arcopilus	P	0.085±0.037a	0.024±0.044	**0.004**	0.475	**0.012**	ns
	PL	0.008±0.007b	0.003±0.004				ns
	PLA	0.005±0.003b	0.016±0.020				ns
	LF	0.002±0.000b	0.031±0.041				ns
